# Is There a Link Between Type 2 Diabetes Mellitus and Negative Symptoms in Schizophrenia? A Scoping Review

**DOI:** 10.3390/brainsci15050499

**Published:** 2025-05-13

**Authors:** István Bitter, Pál Czobor, László Tombor

**Affiliations:** Department of Psychiatry and Psychotherapy, Semmelweis University, 1083 Budapest, Hungary

**Keywords:** schizophrenia, negative symptoms, secondary negative symptoms, diabetes mellitus, impaired glucose metabolism, review

## Abstract

Background/Objectives: Type 2 diabetes mellitus (T2DM) and impaired glucose metabolism are more prevalent among patients with schizophrenia than in the general population. The incidence of T2DM is associated with lifestyle factors that are often influenced by the negative symptoms of schizophrenia; comorbid T2DM may contribute to the reduced life expectancy observed in patients with schizophrenia. The existing literature reveals a scarcity of data regarding the potential causal relationship between T2DM and negative symptoms. Methods: A scoping review was conducted following the PRISMA (Preferred Reporting Items for Systematic Reviews and Meta-Analyses) criteria, utilizing the PubMed database to identify clinical studies investigating the association between T2DM and the negative (but not cognitive) symptom domain of schizophrenia. Subsequently, the reference lists of these identified publications were searched. Results: Seventeen publications were included. There is evidence supporting the association between impaired glucose tolerance and increased negative symptoms in patients with first-episode psychosis, and several studies indicate that poorer glucose metabolic status correlates with more severe negative symptoms. Patients with T2DM and chronic schizophrenia, however, had milder negative symptom scores compared to those without diabetes, although this association was less pronounced than in early disease stages. Conclusions: There is insufficient confirmatory evidence regarding the potential causality of T2DM on the negative symptoms of schizophrenia. Further, preferably prospective studies are needed to explore the complex and potentially causal relationship between T2DM and negative symptoms of schizophrenia. If T2DM were found to have a causal relationship with negative symptoms or to exacerbate pre-existing symptoms, it could lead to significant changes in therapeutic approaches for schizophrenia.

## 1. Introduction

Schizophrenia (SZ) is one of the most prevalent psychiatric disorders that lead to disability primarily due to the high rate of poor functional outcomes associated with the negative and cognitive symptom domains of the disorder [1,2]. Negative symptoms can be categorized as primary and secondary. Primary negative symptoms are considered to be directly related to the pathophysiology of schizophrenia, whereas secondary negative symptoms may have a causal relationship with various factors, including positive symptoms, depression, side effects of antipsychotic treatment (e.g., Parkinsonism), or psychosocial deprivation (e.g., poverty) [3,4]. Although only a limited number of effective therapeutic options exist for the treatment of primary negative symptoms, addressing the underlying causes of secondary negative symptoms may lead to significant clinical improvements [5]. Recent research over the past two decades has begun to differentiate negative symptoms and cognitive impairments as separate domains, culminating in the widely accepted five-domain model of negative symptoms, which includes anhedonia, blunted affect, alogia, asociality, and avolition [6,7,8].

Type 2 diabetes mellitus (T2DM) presents a considerable global disease burden, which is anticipated to increase in the coming decades [9]. The comorbidity of schizophrenia and T2DM is well documented in the literature [10,11,12]. The risk of developing T2DM in individuals with schizophrenia is approximately 2.15 times higher than in the general population [13]. This elevated rate of comorbidity is associated with several factors, including treatment with antipsychotics and the negative symptoms of schizophrenia through their effect on lifestyle [14].

Despite this, there is a paucity of studies exploring the causal relationship between T2DM and negative symptoms of schizophrenia. A review by Perry et al. [15] provided evidence supporting an association between schizophrenia severity and dyslipidemia and indicated a stronger link between negative and cognitive symptoms and glucose markers. Notably, only a minority (three) of the publications directly addressed the negative symptom domain. Furthermore, current comprehensive reviews on negative symptoms in schizophrenia have not included T2DM as a potential causal factor for secondary negative symptoms [8,16].

The primary question guiding this review stems from the authors’ clinical observations that the successful treatment of newly diagnosed comorbid T2DM positively affected the negative symptoms in several patients with schizophrenia. We hypothesize that T2DM may induce secondary negative symptoms in individuals with schizophrenia or exacerbate pre-existing symptoms. The objective of this scoping review is to explore the literature on the relationship between T2DM and negative symptoms in schizophrenia.

## 2. Materials and Methods

A systematic literature search was conducted following the recommendations of the PRISMA (Preferred Reporting Items for Systematic Reviews and Meta-Analyses) Extension for Scoping Reviews [17]. The following search terms were used in the PubMed database on 28 January 2025: (Diabetes mellitus)/(Hyperglycemia)/(Hyperglycaemia)/(HbA1c)/(Glycated hemoglobin)/(Glucose)/(Elevated fasting glucose)/(Prediabetes) AND (Schizophrenia) AND (Negative symptoms). The time scope of the search spanned from 1980 (database inception) to 28 January 2025.

We focused our search on (1) original publications utilizing a study sample of patients with schizophrenia stratified by comorbidity with T2DM (diabetic vs. non-diabetic schizophrenia groups) or (2) original publications covering data on the prevalence and/or glycemic status of schizophrenia populations stratified by negative symptoms and/or (3) original publications investigating the association of serum glucose markers and glycemic status with negative symptoms.

The inclusion criteria for retrieved studies were as follows:

(1) Adolescents or adults (>16 years of age);
(2) A diagnosis of schizophrenia according to the DSM or the ICD classification systems;
(3) Data reported in (pre)diabetic and non-(pre)diabetic schizophrenia/first-episode psychosis groups regarding at least one of the following: (a) negative symptoms assessed by a validated measurement tool, (b) the prevalence rate of T2DM, (c) levels of glycosylated hemoglobin and/or fasting blood glucose levels, (d) glycemic status (including serum markers) of study populations, and (e) the association of negative symptom severity with serum markers of glycemic status;
(4) Publications in English.

Exclusion criteria were as follows:

(1) Diagnosis of schizophrenia was not applied or not made according to the DSM/ICD systems;
(2) No data on the severity of negative symptoms;
(3) Patients with diabetes or patients receiving antidiabetic medication were excluded from participation in the study;
(4) Publications reporting data from schizophrenia study groups with metabolic syndrome or obesity without conducting separate analyses reporting clear data on the presence of a T2DM subgroup (as these groups with obesity or metabolic syndrome groups can be heterogeneous regarding glycemic status);
(5) Publications focused on type 1 diabetes and schizophrenia;
(6) Studies that did not report any serum markers of glycemic status (HbA1c or fasting blood glucose);
(7) Studies reporting negative symptom severity solely in the context of metabolic outcomes.

Titles and/or abstracts of publications retrieved from the search were screened by two authors (I.B. and L.T.) to identify potential papers matching the inclusion and exclusion criteria outlined above. Studies that met the criteria were then examined in the full text. Additionally, the reference lists of the identified papers were individually reviewed for studies that may not have been captured by the initial search. The Scoping Review Checklist has been provided online as Supplementary File ‘Supplementary S1’.

## 3. Results

### 3.1. Study Selection

**Figure 1:**
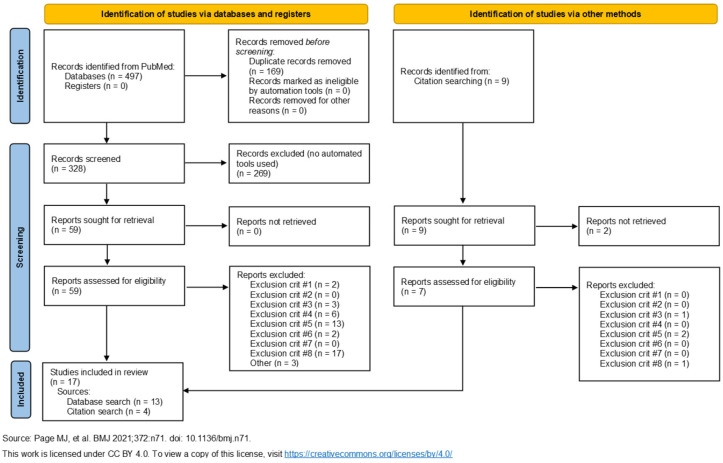
The PRISMA [35] flow diagram. The 17 publications on the left arm of the diagram include the 4 references identified by the citation search.

A total of seventeen studies were selected for inclusion in this review [18,19,20,21,22,23,24,25,26,27,28,29,30,31,32,33,34]. Figure 1 displays the PRISMA flow diagram of the study selection process. Several studies were excluded during the screening process due to irrelevance to the review topic, particularly those focusing on cognitive symptoms. During the full-text review of articles, records were excluded based on the aforementioned exclusion criteria. The publication dates of the included studies range from 2011 to 2024.

Table 1, Table 2 and Table 3 summarize the included studies. Table 1 presents studies that report data on the prevalence rates of T2DM in patient groups characterized by negative symptoms. Table 2 provides an overview of studies that report data on the severity of negative symptoms in patients with schizophrenia who have abnormal glucose metabolism but do not meet the criteria for T2DM (subjects with insulin resistance or prediabetes). Table 3 compares the severity of negative symptoms between patients with schizophrenia and comorbid T2DM (SZ+T2DM) and those without T2DM (SZ−T2DM).

### 3.2. Study Characteristics

Fifteen of the seventeen included studies employed a cross-sectional design. One study [24] was a retrospective cohort study based on the analysis of electronic medical records held by a European healthcare provider; and another study was a retrospective, cross-sectional reanalysis of the results of the CATIE (Clinical Antipsychotic Trials of Intervention Effectiveness) study, focusing on the associations between metabolic alterations and cognitive performance within the original study population. Sample sizes varied from N = 38 to N = 1128. Nine studies described comparisons of negative symptom severity between the SZ+T2DM vs. SZ−T2DM groups [20,21,22,23,25,26,29,30,34]. Six studies reported on negative symptom severity and/or its correlations with glycemic markers in patient groups that did not meet the diagnostic criteria for T2DM but exhibited impaired glucose metabolism (insulin resistance or prediabetes) [18,19,28,31,32,33]. Two studies reported the prevalence rates of T2DM in schizophrenia samples stratified by the presence or severity of negative symptoms [24,27].

### 3.3. Patient Characteristics

The age range of patient samples varied from 16 to 76 years, with a mean age ranging from 26.8 to 64.1 years across studies. Most patients were middle-aged, except for those enrolled in studies focusing on specific patient cohorts, such as late-life schizophrenia (SZ) [20] or first-episode psychosis (FEP) [18,19,27,31,32,33]. Study samples predominantly consisted of male subjects, with the exception of Chen et al. [19], which analyzed a predominantly female sample. All but five studies [18,19,31,32,33] enrolled patients on long-term antipsychotic treatment; in one study, medication status was not reported [27]. Negative symptom severity was measured using the Positive and Negative Syndrome Scale (PANSS) [36] negative subscale (PANSS N) in all but two papers [23,26], where the Brief Psychiatric Rating Scale (BPRS) [37] or the Psychosis Evaluation Tool for Common use by Caregivers (PECC) [38] were utilized. Except for the publications focusing on the prevalence of T2DM among the schizophrenia population [24,27], only five studies [20,22,25,26,30] reported the prevalence rate of T2DM within the total study sample, and four reported the rate of prediabetes or insulin resistance [18,27,31,32]. Only four studies [20,23,25,30] included data on onset and duration of T2DM, as well as treatment and compliance with antidiabetic therapy.

### 3.4. Study Findings

#### 3.4.1. Epidemiological Associations

The retrospective cohort study conducted by Sicras-Mainar et al. [24] utilized electronic records from a healthcare provider to identify schizophrenia (SZ) cases. Of a total of 76,600 persons who sought care in the index year (2011), 1120 patients were identified as eligible for data analysis, possessing complete data on both symptom severity and biological components of metabolic syndrome. Among these, T2DM was analyzed separately. Subjects with schizophrenia were subdivided into two groups based on the presence or absence of at least one negative symptom (‘Presence of negative symptoms’ vs. ‘Absence of negative symptoms’) using the items from the PANSS Marder negative-symptom factor. The authors found that the prevalence of T2DM was significantly higher in the ‘Presence of negative symptoms’ group compared to the ‘Absence of negative symptoms’ group (22.5% vs. 17.1%, respectively, *p* = 0.024).

In a retrospective chart record analysis conducted by Wu et al. [27], an antipsychotic-naïve first-episode schizophrenia cohort was stratified by PANSS subscale scores into four groups: ‘predominantly negative symptoms’ (PANSS N score 20 points or more and PANSS P score 19 or less), ‘with major positive symptoms’ (PANSS P score 20 points or more and PANSS P score 20 or less), ‘both severe negative and positive symptoms’ (PANSS P and PANSS N scores both more than 20), and ‘other’ patients. The authors found that elevated fasting blood glucose levels (prediabetes) were more prevalent in the negative-symptom group compared to the positive-symptom group (31.57% vs. 13.49%, respectively, *p* < 0.0001) along with a trend indicating that insulin resistance was more prevalent in the negative-symptom group compared to the positive-symptom group (61.24% vs. 51.69%, respectively, *p* = 0.058). The results of this section are summarized in Table 1.

**Table 1:** Overview of studies reporting prediabetes or diabetes prevalence rates in patients with schizophrenia with negative symptoms versus control schizophrenia groups.

Author (Year)	Study Type	N SZ ^1^	Study Population	Main Findings
Wu (2024) [27]	Cross-sectional Retrospective	704	Mean age: 27.44 ± 8.91 years Inpatients First-episode psychosis Average DUP ^4^: 14.82 ± 12.99 months	The rate of abnormal glucose level is significantly higher in the NS SZ ^2^ group compared to the PS SZ ^3^ group (31.57% vs. 13.49%). There is a trend that the rate of abnormal insulin resistance is higher among NS SZ ^2^ than PS SZ ^3^ (61.24% vs. 51.69%). Fasting blood glucose (r = 0.229, *p* < 0.0001), fasting insulin (r = 0.221, *p* < 0.0001), and HOMA-IR ^5^ (r = 0.259, *p* < 0.0001) exhibited moderate strength and a significant positive correlation with the PANSS N ^6^ subscale score.
Sicras-Mainar (2015) [24]	Non-interventional Retrospective Cohort study	1120	Mean age: 46.8 ± 13.8 years 58.4% male SZ ^7^ outpatients NS group: 52.5% Mean number of medications including AP ^8^: 3.1 ± 1.9	The prevalence of T2DM ^9^ was higher in the NS SZ ^1^ group than the non-NS SZ group (22.5% vs. 17.1%, respectively, *p* = 0.024).

^1^ N SZ—number of subjects with schizophrenia, ^2^ NS SZ—schizophrenia with prominent negative symptoms, ^3^ PS SZ—schizophrenia with prominent positive symptoms, ^4^ DUP—duration of untreated psychosis, ^5^ HOMA-IR—homeostasis model assessment of insulin resistance, ^6^ PANSS N—Positive and Negative Syndrome Scale Negative subscale, ^7^ SZ—schizophrenia, ^8^ AP—antipsychotic, ^9^ T2DM—type 2 diabetes mellitus.

#### 3.4.2. First-Episode Psychosis

Although prediabetes and insulin resistance are not the primary focus of this review, it is important to note that several studies [18,27,31,32,33] investigating patients with who were medication-naïve with first-episode schizophrenia (SZ) found associations between glycemic blood markers and negative symptoms of schizophrenia. An overview is provided in Table 2. Prediabetes is an intermediate stage of dysglycemia characterized by one of the following: (1) elevated fasting blood glucose (FBG), (2) elevated glycated hemoglobin (HbA1c), or (3) increased 2 h post-load blood glucose (impaired glucose tolerance, IGT) [39]. Prediabetes is preceded by insulin resistance, and the Homeostasis Model Assessment of Insulin Resistance (HOMA-IR) is the most commonly used method for its quantitative estimation [40].

In a cross-sectional study by Chen et al. [18], the prevalence rate of IGT was found to be 25% among patients with SZ (n = 172) compared to 0% in controls (n = 31). Furthermore, SZ with IGT exhibited significantly more severe negative symptoms compared to those without IGT (PANSS N score 21.7 ± 9.2 vs. 19.0 ± 7.6, respectively, *p* = 0.048). Another cross-sectional analysis indicated that fasting blood glucose, fasting insulin, and HOMA-IR had a modest but significant positive correlation with the PANSS N subscale score in a medication-naïve first-episode psychosis population. Higher FBG, serum insulin, and HOMA-IR were associated with more severe negative symptoms [27]. Two publications by the same research group found a trend indicating that male patients with SZ and IGT had more severe negative symptoms than patients without IGT (24.40 ± 9.86 vs. 20.57 ± 8.57, *p* = 0.09) [32]; however, this trend was not observed in female patients with first-episode SZ [33]. Another study reported a significant positive correlation between fasting blood glucose levels and the PANSS N scale (r = 0.13, *p* = 0.03). One study [31] found that glucose metabolism markers and insulin resistance were unrelated to the PANSS N score but were associated with the positive symptom domain, indicating that higher HOMA-IR was linked to more severe positive symptoms (PANSS P score) [19]. In a cross-sectional study with a modest total population size (N = 46), fasting blood glucose and the PANSS N score were found to be unrelated [28]. For an overview of these studies, please refer to Table 2.

**Table 2:** Overview of studies reporting findings from first-episode schizophrenia groups with and without prediabetes or insulin resistance.

Author (Year)	Study Type	N (SZ+PreD)	N (SZ−PreD)	Patient Characteristics	Findings
Chen (2016) [18]	C-S	43	129	Mean age: 28.7 ± 9.9 Age range: 18–45 years 48.25% male First-episode psychosis Inpatients + follow-up Medication naïve Duration of psychosis <60 months. IGT rate 25% compared to 0% in HC.	The SZ+IGT group had a significantly higher PANSS N score than SZ−IGT (21.7 ± 9.2 vs. 19.0 ± 7.6).
Chen (2013) [19]	C-S	49	N/A	Mean age: 26.8 ± 8.1 Age range: 16–45 years 28.6% male First-episode psychosis Inpatients Medication naïve or short-term (<2 weeks) AP treatment (57% vs. 43%, respectively)	Insulin resistance and glycemic parameters were not associated with negative symptom severity.
Wysokinski (2013) [28] *	C-S	20	26	Mean age: 31.7 ± 10.09 Age range: 16–45 years 76% male Inpatients with SZ Antipsychotic treatment	No association was found between the PANSS N scale score and the presence of elevated FBG.
Lang (2021) [31]	C-S	430	N/A	Mean age: 32.72 ± 11.26 years 48.4% male Inpatients with first-episode SZ Duration of symptoms < 5 years Antipsychotic-naïve Partial overlap with the Li (2021) [22] study cohort	The prevalence of elevated HbA1c and elevated serum insulin was significantly higher in the patient group than in healthy controls (25.6% vs. 10.8%, *p* < 0.0001, and 9.1% vs. 0.9%, *p* < 0.0001, respectively). In the patient group, fasting glucose level was significantly associated with the PANSS N score (r = 0.13, *p* = 0.03).
Li (2022) [32]	C-S	20/12 **	63/49 **	First-episode AND chronic SZ Inpatients 100% male Mean age **: 26.90 ± 9.12/45.08 ± 6.38 years Antipsychotic medication FEP group: <2 weeks duration Chronic group: 370.03 ± 179.68 mg/day CPZ dose	T2DM prevalence is 4.9% among chronic SZ vs. 0% in the FEP group. FBG is significantly higher in FEP vs. chronic SZ (4.98 ± 1.04 vs. 4.52 ± 0.80 mmol/L, *p* < 0.01). Trend in the FEP group that SZ+PreD had more severe negative symptoms than SZ−PreD (PANSS N 24.40 ± 9.86 vs. 20.57 ± 8.57, *p* = 0.09). The SZ+PreD chronic SZ group had significantly more severe negative symptoms than the SZ−PreD group (24.76 ± 9.14 17.07 ± 5.28, *p* = 0.003).
Li (2025) [33]	C-S	43	129	First-episode SZ Inpatients 48.3% male Mean age: 28.90 ± 9.97 years Antipsychotic medication < 2 weeks duration	Trend in the FEP group that male patients with SZ+PreD had more severe negative symptoms than male patients with SZ−PreD (PANSS N 24.40 ± 9.86 vs. 20.57 ± 8.57, *p* = 0.09). This trend is missing in female patients (PANSS N 17.81 ± 7.81 vs. 18.24 ± 6.34, SZ+PreD vs. SZ−PreD, NS)

* Study groups were subjects with schizophrenia with vs. without elevated fasting blood glucose. ** First-episode schizophrenia/chronic schizophrenia groups, respectively. SZ+PreD—schizophrenia with prediabetes, SZ−PreD—schizophrenia without prediabetes, C-S—cross-sectional, SZ+IGT—schizophrenia with IGT, PANSS N—Positive and Negative Syndrome Scale Negative subscale, SZ−IGT—schizophrenia without IGT, IGT—impaired glucose tolerance, FBG—fasting blood glucose, T2DM—type 2 diabetes mellitus, FEP—first-episode psychosis, SZ—schizophrenia, CPZ—chlorpromazine, AP—antipsychotic, HC—healthy controls, NS—not significant.

#### 3.4.3. Chronic Schizophrenia

Studies comparing symptom severity in patients with schizophrenia and T2DM (SZ+T2DM) to those without (SZ−T2DM) found no significant differences between the study groups [20,21,22,25,29,30,34] (see Table 3 for a summary of findings). However, with the exception of two publications [26,34], patients with SZ+T2DM exhibited numerically lower PANSS N scores across all studies compared to patients with SZ−T2DM. One notable exception is a study [26] that utilized the Psychosis Evaluation Tool for Common use by Caregivers (PECC) scale for measuring symptom severity, where patients with SZ+T2DM had numerically higher scores, although this difference did not reach statistical significance. It is important that all of these studies were cross-sectional and analyzed data of chronic schizophrenia inpatients on maintenance antipsychotic therapy. This maintenance antipsychotic therapy primarily involved monotherapy with second-generation antipsychotics, including clozapine, in six [20,22,26,29,30,34] of the nine studies reviewed. The proportion of the total patient population (SZ+T2DM and SZ−T2DM) receiving clozapine ranged from 11 to 51% across the study cohorts (see Table 3).

Furthermore, most studies did not report significant differences in positive symptom severity, general symptom severity, or the PANSS total score between study groups. Publications comparing SZ+T2DM and SZ−T2DM generally did not provide data on the age of onset, duration, severity, treatment of T2DM, or about therapeutic compliance with antidiabetics or non-pharmacological treatment options, with the exception of Huo et al. [20], Takanayagi et al. [25], and Ogawa et al. [23]. In the study by Huo, elderly patients with schizophrenia had an average T2DM duration of 57.44 ± 67.70 months, and 84% of the diabetic population was on stable antidiabetic medication. Takanayagi et al. reported that approximately 40% of the analyzed patients with SZ+T2DM were not receiving antidiabetic therapy. Among those on maintenance therapy, the patients used oral antidiabetics alone or in combination with insulin. The serum levels of the Hba1c for compliant versus non-compliant study groups were not discussed in detail, leaving uncertainty about the effectiveness of diabetes management. Ogawa et al. [23] investigated glycated hemoglobin levels in outpatients with schizophrenia and type 2 diabetes mellitus (SZ+T2DM) who were on maintenance antidiabetic therapy, with 81.6% of participants using oral antidiabetics. The median Brief Psychiatric Rating Scale (BPRS) negative factor [41] value was utilized to categorize patients into low- and high-intensity negative symptom groups. The authors concluded that the severity of negative symptoms did not influence treatment efficacy, as serum HbA1c levels did not differ between study groups. For an overview, please refer to Table 3.

**Table 3:** Overview of studies reporting group differences between schizophrenia groups with (SZ+T2DM) and without type 2 diabetes (SZ−T2DM).

Author (Year)	Study Type	N (SZ+T2DM)	N (SZ−T2DM)	Patient Characteristics	Findings
Li (2021) [22]	Cross-sectional	54	418	Mean age 47.22 years 87% male Chronic SZ (mean duration: 23.83 ± 8.50 years) Inpatients Antipsychotic monotherapy (mean CPZ dose: 457.14 ± 394.93 mg/day) CLO therapy: 51% Other SGA therapy: 25% T2DM prevalence: 11.4%	PANSS N subscale scores did not differ significantly between SZ+T2DM vs. SZ−T2DM groups (20.80 ± 6.64 vs. 21.84 ± 7.16, respectively). SZ+T2DM had superior cognitive performance and higher BDNF levels than SZ−T2DM.
Huo (2020) [21]	Cross-sectional	140	1049	Mean age 48.51 ± 10.09 years (age range 16–76 years) 79% male Early-stage and chronic SZ (illness duration 1–19 years) Inpatients Antipsychotic therapy (mean CPZ doses 479.93 ± 526.89 vs. 438.29 ± 384.01 mg/day SZ+T2DM vs. SZ−T2DM) CLO therapy: N/A SGA therapy: 75% T2DM prevalence: 12.53%	PANSS N subscale scores did not differ significantly between SZ+T2DM vs. SZ−T2DM groups (22.03 ± 8.27 vs. 22.79 ± 8.4, respectively). SZ+T2DM had more severe positive symptoms than SZ−T2DM.
Huo (2021) [20]	Cross-sectional	73	216	Mean age 64.11 ± 3.26 years (age range 60–76 years) 72% male Elderly (>60 years of age) Chronic SZ (Mean illness duration 35.34 ± 9.15 years) Inpatients Antipsychotic therapy (mean CPZ dose 363.61 ± 285.65 mg/day) CLO therapy: 23% Other SGA therapy: 59% Prevalence of T2DM: 23.5% Duration of diabetes: 57.44 ± 67.70 months Stable hypoglycemic medication: 84%	PANSS N subscale scores did not differ significantly between elderly SZ+T2DM vs. SZ−T2DM groups (22.85 ± 6.33 vs. 24.09 ± 7.42, respectively). Elderly patients with SZ+T2DM had more severe positive symptoms than those with SZ−T2DM.
Zhang (2011) [30]	Cross-sectional	46	160	Age range 25–70 years 68% male Chronic SZ (mean disease duration 28.6 ± 9.5 vs. 25.6 ± 9.8 SZ+T2DM vs. SZ−T2DM, respectively) Inpatients CLO therapy: 100% Mean CLO dose: 180.1 ± 95.1 vs. 171.2 ± 83.9 SZ+T2DM vs. SZ−T2DM, respectively, Treatment duration with CLO: 70.1 ± 57.8 vs. 62.0 ± 59.7 SZ+T2DM vs. SZ−T2DM, respectively, Prevalence of T2DM 22.3% No data on T2DM characteristics or treatment	PANSS N subscale scores did not differ significantly between SZ+T2DM vs. SZ−T2DM groups (21.1 ± 8.4 vs. 21.5 ± 7.7, respectively). The SZ+T2DM vs. SZ−T2DM groups did not differ in PANSS total score or other PANSS subscales. Treatment with clozapine was associated with a high risk of T2DM.
Zhang (2015) [29]	Cross-sectional	101	162	Age range 40–68 years) 73% male Chronic SZ (>5 years of duration) Inpatients Antipsychotic therapy (mean CPZ dose 415.3 ± 370.8 mg/day) CLO therapy: 44% Other SGA therapy: 32% No data on T2DM characteristics or treatment	PANSS N subscale scores did not differ significantly between SZ+T2DM vs. SZ−T2DM groups. Female patients had lower negative symptom severity than male patients.
Takayanagi (2012) [25]	CATIE SZ study	161	1128	Age range 18–65 years) 75% male Chronic SZ (Illness duration 18.8 ± 10.1 vs. 13.6 ± 10.5 years SZ+T2DM vs. SZ−T2DM, respectively) Antipsychotic therapy (mean CPZ doses not reported for study groups) CLO therapy: 0% Duration of T2DM is unknown. Treatment for T2DM: most SZ+T2DM 40% did not take medication for T2DM.	PANSS N subscale scores did not differ significantly between SZ+T2DM vs. SZ−T2DM groups (19.3 ± 6.0 vs. 20.0 ± 6.4, respectively). Total PANSS score did not differ between SZ+T2DM vs. SZ−T2DM.
Ogawa (2011) [23]	Cross-sectional	19	19	Mean age: 53.9 ± 8.5 (range: 37–68 years) 60.5% male Age at T2DM diagnosis: 46.2 ± 9.4 (range: 24–66 years) Antipsychotic treatment: 100% CLO therapy: N/A SGA therapy: 42% Outpatients T2DM treatment: 100% OAD: 81.6%	Hba1c level did not differ between low and high negative symptom groups (Negative symptoms subscale: 4 points = no symptoms [n = 14] vs. ≥5 points [n = 24]). Hba1c level did not differ between low- and high total BPRS and positive subscale groups.
Vancampfort (2013) [26]	Cross-sectional	10	86	Age range: 18–65 years Mean age: 40.3 ± 10.9 vs. 34.5 ± 10.5 years SZ+T2DM vs. SZ−T2DM, respectively, 58% male All but one on medication therapy, including antipsychotics CLO therapy: 11% Other SGA therapy: 50%	Negative symptom severity was more severe in SZ+T2DM vs. SZ−T2DM, measured with the PECC N subscale (12.6 ± 5.9 vs. 9.8 ± 4.7, respectively). The difference did not reach statistical significance.
Yan (2023) [34]	Cross-sectional Multicenter	136	852	Age range: 17–70 years 68.7% male Inpatients Chronic SZ Mean SZ duration: 21.71 ± 11.90 years Antipsychotic treatment Mean CPZ dose: 396.76 ± 808.57 mg/day CLO therapy: 34% Other SGA therapy: 56%	No statistically significant difference is detected in the PANSS N scale score in men or women SZ patients (Male SZ+T2DM vs. SZ−T2DM 22.08 ± 6.22 vs. 21.52 ± 6.86, NS, and Female SZ+T2DM vs. SZ−T2DM 21.65 ± 5.69 vs. 22.45 ± 7.78, NS).

SZ+T2DM—schizophrenia with type 2 diabetes mellitus, SZ−T2DM—schizophrenia without type 2 diabetes mellitus, SZ—schizophrenia, CPZ—chlorpromazine, T2DM—type 2 diabetes mellitus, PANSS N—Positive and Negative Syndrome Scale Negative subscale, BDNF—brain derived neurotrophic factor, CLO—clozapine, OAD—oral antidiabetic, PECC N—Psychosis Evaluation Tool for Common use by Caregivers Negative subscale, BPRS—Brief Psychiatric Rating Scale, NS—not significant.

## 4. Discussion

### 4.1. Summary of Key Findings

The primary finding of this review is the notable lack of prospective studies examining the causal role of T2DM on negative symptoms in schizophrenia. We also found that the results of existing cross-sectional studies are heterogeneous and controversial. Studies investigating the epidemiological association between T2DM and schizophrenia negative symptoms found that in patient groups characterized by more severe negative symptomatology, the prevalence of T2DM was higher than in comparison with schizophrenia patient groups. Additionally, the literature suggests that the association of T2DM may differ at various stages of schizophrenia. While most studies investigating populations with first-episode schizophrenia (FEP) found supporting evidence that patients with impaired glucose metabolism exhibited more severe negative symptom scores compared to metabolically healthy patients, the literature appears to contradict this finding in chronic schizophrenia, as there is no significant difference in the severity of negative symptoms between patients with schizophrenia and type 2 diabetes mellitus (SZ+T2DM) and those without (SZ−T2DM). Moreover, patients with SZ+T2DM exhibited numerically lower PANSS N scale scores than patients with SZ−T2DM in most studies.

### 4.2. Interpretation of the Findings

#### 4.2.1. Epidemiological Associations

Regarding the epidemiological association of T2DM and negative symptoms of schizophrenia, it is of note that the definition of the ‘negative symptom group’ varied between studies, and several factors may contribute to this epidemiological association, including but not limited to sociodemographic status, age, gender, and the ethnic composition of study groups [42,43], and the existence and duration of current antipsychotic use, as these factors influence the risk of T2DM [44,45,46]. The elevated T2DM risk among patients with negative symptoms is partially consistent with the literature, indicating that unfavorable metabolic outcomes are influenced by schizophrenia symptoms [14]; however, the direction of this relationship contrasts with the hypothesis posed in our work.

#### 4.2.2. Potential Common Pathomechanisms of Schizophrenia and Type 2 Diabetes

Several factors have been implicated as contributors to the association between negative symptoms of schizophrenia and T2DM.

There is some evidence in the literature suggesting an interaction between peripheral glucose levels and cerebral dopamine transmission. For instance, striatal dopamine D2/3 receptor availability has been found to be inversely associated with body mass index (BMI) in a group of individuals with obesity [47]. Another study reported that systemic dopamine depletion impaired insulin-mediated glucose uptake [48]. According to the dopamine hypothesis of schizophrenia [49], excessive dopamine activity in subcortical regions is linked to positive symptoms, whereas dopamine depletion in the prefrontal cortices contributes to the negative symptoms [50]. Intermittent theta-burst stimulation of the left dorsolateral prefrontal cortex in patients with chronic schizophrenia with predominantly negative symptoms was shown to improve both negative symptom scores and elevated fasting blood glucose levels, highlighting a possible connection between prefrontal dopamine function and glucose regulation. This relationship was further supported by a complex animal study by the same group. Surgical transection and the chemogenic inhibition of the pathways between the ventral tegmental area and the prefrontal cortex resulted in glucose intolerance in mice. Similarly, local infusion of dopamine receptor antagonists into the medial prefrontal cortex produced identical metabolic effects [27]. The hypothesized link between dopaminergic transmission and glucose homeostasis is further supported by findings from genetic studies indicating a shared genetic background of schizophrenia and type 2 diabetes [51,52,53].

An important factor associated with both schizophrenia and diabetes is the alteration of peripheral, and subsequently central, levels of pro-inflammatory and anti-inflammatory cytokines. Peripheral levels of Interleukin-1β (IL-1β), Interleukin-6 (IL-6), and tumor necrosis factor-α (TNF-α) are elevated during first-episode psychosis and psychotic relapses in schizophrenia [54,55], as well as in the pathogenesis [56] and complications of type 2 diabetes [57,58]. Conversely, levels of anti-inflammatory cytokines, such as Interleukin-10 (IL-10), tend to be reduced in both conditions. Elevated concentrations of cerebrospinal fluid IL-1 and IL-6 were reported from the cerebrospinal fluid of patients with chronic schizophrenia [59,60]. These peripheral pro-inflammatory cytokines can access the central system through various pathways, ultimately leading to microglia activation. This, in turn, induces a local pro-inflammatory response in neural tissue, alters tryptophan metabolism, increases oxidative stress, and activates the endothelium in cerebral blood vessels. Disrupted tryptophan metabolism results in increased production of kynurenic acid and quinolinic acid. Of these, kynurenic acid—an NMDA receptor antagonist—is hypothesized to contribute to the positive symptom domain of schizophrenia, while quinolinic acid is associated with increased oxidative stress and may promote neurodegeneration observed in chronic schizophrenia. Activated microglia also produce glutamate, which is excessively released into the extracellular space under a pro-inflammatory condition. Elevated glutamate levels in the central nervous system induce excitotoxicity, further contributing to neuronal loss. These mechanisms potentially explain the more severe negative symptoms of schizophrenia in FEP patients with schizophrenia with comorbid diabetes [61].

The novel approach of analyzing the gut microbiome has revealed that certain alterations of gut bacterial genera can disrupt the balance between energy intake and expenditure, leading to weight gain. This, in turn, increases the risk of type 2 diabetes. Moreover, these bacteria produce pro-inflammatory molecules, such as lipopolysaccharides and pro-inflammatory cytokines, including IL-6 and TNF-α, which may exacerbate the processes previously discussed in relation to both schizophrenia and glucose homeostasis [62,63].

#### 4.2.3. Findings in Chronic Schizophrenia

A potential confounding factor that may explain the less severe negative symptoms in chronic SZ+T2DM is antidiabetic treatment. The most common first-line pharmacotherapy for T2DM, in the absence of severe somatic complications, is metformin [64]. Preclinical studies indicate that metformin can cross the blood–brain barrier and possesses neuroprotective and anti-inflammatory properties. These effects are achieved by decreasing the blood-brain-barrier permeability, inhibiting the microglia and pro-inflammatory cytokines, most notably tumor necrosis factor-alpha (TNFα), interleukin-1β, and interleukin-6, while promoting anti-inflammatory cytokines such as interleukin-10 and insulin-like growth factor 1 (IGF-1). Additionally, metformin activates AMP-activated protein kinase (AMPK), which is typically downregulated in metabolic disturbances such as chronic hyperglycemia in pre(diabetic) patients [53,54]. A recent meta-analysis of randomized controlled trials investigating the efficacy of add-on metformin in antipsychotic-treated SZ populations for reducing antipsychotic-induced weight gain found that cognitive symptoms, and to a lesser extent other symptom dimensions of schizophrenia, slightly improved during the treatment period covered by the trials (24–36 weeks). The authors noted that the trial durations may not have been long enough for the hypothesized neuroprotective effects of metformin to manifest [65].

Additionally, recent meta-analyses concerning the use of add-on topiramate with antipsychotic therapy indicate that topiramate reduces antipsychotic-induced weight gain while also having a beneficial effect on overall schizophrenia severity. However, the effects reported in these studies extend beyond the negative symptom domain to encompass the overall severity of schizophrenia [66,67]. A randomized clinical trial found that add-on pioglitazone to risperidone was superior to placebo in the reduction in negative and overall symptom severity within eight weeks [68]. In contrast, intranasal insulin [69] or the novel glucagon-like peptide-1 receptor agonist exenatide [70] failed to meet satisfactory study endpoints.

As discussed earlier in the Results Section, most publications reviewed did not detail the characteristics of T2DM (e.g., duration, rates of complications, and the efficacy of glycemic control), antidiabetic treatment (e.g., type of pharmacologic and non-pharmacologic treatment), or compliance with antidiabetic treatment. Therefore, the data leave uncertainty about the extent to which antidiabetic treatment influenced the slightly lower negative symptom scores in SZ+T2DM compared to SZ−T2DM. However, most studies included inpatient samples, where treatment adherence is likely better than among outpatients. Furthermore, the recent literature indicates that patients with schizophrenia are more adherent to diabetes treatment than their counterparts without schizophrenia [71] with adherence rates ranging from 51 to 85% [72]. This suggests that the potential symptomatic effects of antidiabetic medications on schizophrenia symptoms should not be overlooked in the reviewed samples.

In addition to the use of antidiabetic medications, physical activity plays an important role in the management of diabetes and prediabetes. The spectrum of physical activities includes aerobic exercise, which involves repeated movements of large muscle groups to increase energy expenditure, resistance (strength) training to reduce fat mass, flexibility training to improve range of motion around joints, and balance training to improve gait stability and prevent falls. According to a recent position statement of the American Diabetes Association (ADA) [73], physical exercises are recommended for individuals with diabetes or prediabetes. Specifically, the statement emphasizes the efficacy of aerobic and resistance exercises in enhancing glycemic control across all age groups. Additionally, it highlights the significance of flexibility and balance exercises for elderly adults. The recommended weekly minimum of 150 min of exercise should be distributed across at least three days, with no more than two consecutive days between each session. A large multicenter prospective study [74] with over 12 years of follow-up found that physical activity alone was effective in reducing the transition from prediabetes to diabetes. The intervention was particularly effective in individuals with low baseline activity levels, who exhibited the most significant reduction in the incidence of type 2 diabetes. Compliance with recommended lifestyle changes for patients with T2DM (diet, increase in physical activity, etc.) and their impact on T2DM were not reported in the reviewed papers, and therefore, it is not possible to assess their potential influence on the severity of negative symptom scores observed in subjects with SZ+T2DM.

It should also be considered that factors independent of T2DM characteristics or diabetes treatment may help explain the apparent contradiction between FEP and chronic schizophrenia cohorts, specifically, the slightly lower negative symptom scores observed in SZ+T2DM compared to SZ−T2DM.

The so-called survivorship bias refers to the tendency for only individuals with better prognoses to be included in studies, as they are the ones who survive long enough to be analyzed. The issue is often discussed in the context of increased mortality in individuals with schizophrenia [75,76,77]. The reviewed literature of chronic schizophrenia cohorts in our work may have included patients with more favorable outcomes in terms of physical health and/or overall survival. Since severe or uncontrolled physical illnesses were exclusion criteria in most of the reviewed studies, it is plausible that data from patients with schizophrenia with serious T2DM complications were not included. This exclusion could potentially confound the observed findings. Similarly, suicidality may have acted as an additional confounding factor in the reviewed cross-sectional studies. As data from patients who did not survive are unavailable, and these individuals likely had more severe clinical profiles, their exclusion could bias the results.

It should also be noted that patients with chronic schizophrenia with longer disease duration included in cross-sectional studies may represent a subgroup with better adaptation to their chronic illness. The concept of adaptation to chronic illness [78] encompasses a complex process involving psychosocial adjustment through the effective use of beneficial coping mechanisms and enhanced resilience, environmental adaptation, and better caregiver support. However, most studies in our review included inpatient samples of chronic patients with schizophrenia. In these cases, it is unclear why the patients were hospitalized (e.g., due to acute episodes or inability to live independently) or how long they had been hospitalized, as such data were not reported. Therefore, it remains uncertain whether these patients truly represent a cohort with better adaptive capacities.

It is also important to note that the disease course of patients with chronic schizophrenia may follow different symptom trajectories, which are often overlooked in cross-sectional studies. These variations represent a potential source of heterogeneity and may confound interpretations. Distinct trajectories of positive symptoms (e.g., relapsing vs. continuous) have been recognized for a long time. More recently, different trajectories of negative symptoms have also been described. For example, in a 20-year longitudinal study, Starzer et al. [79] identified two distinct negative symptom trajectories: ‘symptom remission’ and ‘continuous symptoms’. Similarly, in a 6-year follow-up study, Habtewold et al. [80] reported three trajectories labeled as ‘low’, ‘high-increased’, and ‘high-decreased’. It is plausible that chronic schizophrenia subjects in the reviewed studies also followed different courses of the negative symptoms prior to enrollment; however, due to the cross-sectional design, such longitudinal patterns remain unobserved. Both Starzer and Habtewold identified trajectories (‘symptom remission’ and ‘high-decreased’) characterized by a marked attenuation of negative symptoms over time following an initially high symptom level. This may also help explain our finding of lower negative symptom severity in the chronic schizophrenia cohorts.

### 4.3. Clinical and Therapeutic Implications

If our hypothesis regarding the potential role of T2DM in influencing the severity of negative symptoms in schizophrenia holds true, it may suggest the need for the re-evaluation of treatment and long-term care strategies for patients with schizophrenia. Implementing early, targeted screening programs for type 2 diabetes, along with both non-pharmacological and pharmacological interventions, could help reduce the risk of somatic complications and potentially increase life expectancy.

Recent reviews have reported that lifestyle interventions focused solely on regular physical activity (‘exercise therapy’) in patients with schizophrenia were associated with reduced symptom severity at the study endpoints in most of the analyzed publications [81,82]. However, the studies included in these reviews often had small sample sizes, and the results were inconsistent regarding which symptom domain showed the greatest improvement during the intervention. A well-replicated finding was the reduction in general psychopathology (as measured by PANSS G subscale or total PANSS score). A smaller yet significant effect, ranging from small to moderate effect size, was observed for negative symptoms (see meta-analyses [83,84,85]). The hypothesized mechanism of effect underlying the improvement of psychopathology is that regular physical activity enhances neuroplasticity. This hypothesis is further substantiated by imaging studies that reported volumetric increases in specific brain regions, such as the hippocampus [86,87]. Exercise therapy, as a component of a ‘treatment package’ that includes non-pharmacological and pharmacological elements, is likely to be well tolerated by patients with schizophrenia and may have additional benefits such as symptom reduction, improved quality of life, and enhanced global functioning [5,88].

### 4.4. Limitations and Future Directions

There are several limitations to our study. First, most studies covered in this review employed a cross-sectional design, making it impossible to test the hypothesis based on the authors’ clinical observations. To rigorously test the hypothesis, further prospective studies are needed. Second, the patient population primarily comprised inpatients, which limits the generalizability of the findings. Third, the chronic schizophrenia populations received long-term and stable antipsychotic treatment, primarily second-generation antipsychotics including clozapine. The chronic exposure to antipsychotics could confound the findings of a hypothesized link between negative symptoms of schizophrenia and type 2 diabetes. Fourth, the clinical data on T2DM, including pharmacological and non-pharmacological treatment, adherence to therapeutic interventions, and efficacy of glycemic control, were not detailed in the studies covered by this review. These are all potential confounding factors and should be analyzed in detail in future studies. Fifth, most publications reviewed in our work investigated Asian populations. The prevalence rate of T2DM varies between cultures due to complex environmental and biological factors. Specifically, Asian individuals have a higher susceptibility to T2DM even with normal BMI. This might limit the generalizability of our results. Sixth, patients described in the literature as ‘first-episode schizophrenia subjects’ may represent a more heterogeneous population than those with chronic schizophrenia. Seventh, the negative symptom rating scales used in the cited studies do not differentiate between primary and secondary negative symptoms, vary in their sensitivity to different dimensions of negative symptoms (such as emotional expression and motivation/pleasure), and are all susceptible to large placebo responses.

To further investigate the potential link between the negative symptoms of schizophrenia and type 2 diabetes, a combination of longitudinal and cross-sectional study designs is warranted. Such an approach would help mitigate biases related to survivorship and illness adaptation, while also accounting for the heterogeneity of schizophrenia symptom trajectories, antipsychotics, different stages of diabetes, or demographic variables such as ethnicity.

## 5. Conclusions

Based on the current literature, the potential association between T2DM and schizophrenia may vary throughout the course of the disease. Regular and early diabetes screening is essential for patients living with schizophrenia to identify dysglycemia or T2DM. The early detection and treatment of T2DM may not only enhance life expectancy and quality of life for patients with schizophrenia but may also improve the clinical course of the disorder, partially by reducing secondary negative symptoms potentially associated with T2DM. Further longitudinal studies are needed to address the link between T2DM and primary and secondary negative symptoms in schizophrenia.

## Data Availability

No new data were created during the review process.

## Supplementary Materials

The following supporting information can be downloaded at https://www.mdpi.com/article/10.3390/brainsci15050499/s1, Supplementary S1: PRISMA Scoping Review checklist.

## Abbreviations

The following abbreviations are used in this manuscript:

SZ	Schizophrenia
T2DM	Type 2 diabetes mellitus
SZ+T2DM	Schizophrenia with type 2 diabetes mellitus
SZ−T2DM	Schizophrenia without type 2 diabetes mellitus
DSM	Diagnostic and Statistical Manual of Mental Disorders
ICD	International Classification of Diseases
PRISMA	Preferred Reporting Items for Systematic Reviews and Meta-Analyses
CATIE	Clinical Antipsychotic Trials of Intervention Effectiveness
PANSS	Positive and Negative Syndrome Scale
BPRS	Brief Psychiatric Rating Scale
PECC	Psychosis Evaluation Tool for Common Use by Caregivers
CPZ	Chlorpromazine
CLO	Clozapine
OAD	Oral antidiabetic
IGT	Impaired glucose tolerance
IL-1β	Interleukin-1β
IL-6	Interleukin-6
IL-10	Interleukin-10
FBG	Fasting blood glucose
HOMA-IR	Homeostatic Model Assessment for Insulin Resistance
OGTT	Oral glucose tolerance test
PreD	Prediabetes
SZ+PreD	Schizophrenia with prediabetes
SZ−PreD	Schizophrenia without prediabetes
NS SZ	Schizophrenia with prominent negative symptoms
PS SZ	Schizophrenia with prominent positive symptoms
DUP	Duration of untreated psychosis
FEP	First-episode psychosis
AP	Antipsychotic
HC	Healthy controls
NS	Not significant
TNF-α	Tumor necrosis factor-α
N/A	Not applicable
